# Spaceflight and sport science: Physiological monitoring and countermeasures for the astronaut–athlete on Mars exploration missions

**DOI:** 10.1113/EP091595

**Published:** 2025-04-08

**Authors:** Luke DeVirgiliis, Nicholas J. Goode, Kurt W. McDowell, Kirk L. English, Robert Novo, Virina Botros, Ginika Agwu, Jessica M. Scott, Lori L. Ploutz‐Snyder

**Affiliations:** ^1^ Department of Exercise Science and Sport Science East Tennessee State University Johnson City Tennessee USA; ^2^ Department of Sport and Exercise Science Milligan University Milligan Tennessee USA; ^3^ Department of Medicine Memorial Sloan Kettering Cancer Center New York New York USA; ^4^ Albert Dorman Honors College New Jersey Institute of Technology Newark New Jersey USA; ^5^ Department of Medicine CUNY School of Medicine New York New York USA; ^6^ School of Kinesiology University of Michigan Ann Arbor Michigan USA

**Keywords:** athlete monitoring, countermeasures, human/environmental and exercise physiology, spaceflight, sport science

## Abstract

Long‐duration spaceflight impacts essentially every system in the human body, resulting in multisystem deconditioning that might impair the health and performance of crewmembers, particularly on long‐duration exploration missions to Mars. In this review, we apply the sport science model of athlete monitoring, testing and training to astronauts; tactical athletes, whose occupation includes physically demanding tasks. We discuss exploration‐specific physiological monitoring modalities and provide a brief historical overview of physiological countermeasures to spaceflight. Finally, we suggest countermeasures to protect exploration crew health and performance, including targeted preflight and in‐flight exercise training, in‐flight exercise hardware and adjunct individualized nutrition and sleep considerations. Mars exploration missions will be exemplars of the astronaut–athlete paradigm. An integrated approach to physiological monitoring and countermeasures will maximize the likelihood of exploration mission success.

## Abstract (highlights)

- **What is the topic of this review?**
  This review includes both a historical overview and recommendations for future physiological monitoring and countermeasures for long‐duration spaceflight. We apply a sport science model of athlete development to spaceflight crewmembers, who can be viewed as tactical athletes performing physically demanding occupational tasks.
- **What advances does it highlight**?
  Physiological interventions that span pre‐ and in‐flight settings and combine novel and individualized exercise, nutrition and sleep considerations will be needed to protect crewmember health and performance for a Mars mission. We advocate a sport science approach to physiological monitoring and countermeasures for the astronaut–athletes serving as crewmembers on Mars exploration missions.

## 1 INTRODUCTION

Prolonged spaceflight in the absence of appropriate countermeasures is associated with a litany of physiological adaptations that affect virtually every system in the human body (Scott et al., 2023). For example, unloading‐induced cardiovascular changes include reductions in plasma volume, red blood cell mass and count, left ventricular mass, maximal stroke volume, cardiac output and aerobic capacity (Gallo et al., 2020; Moore et al., 2014), and an associated alteration in O_2_ uptake kinetics (Hoffmann et al., 2016). Neuromuscular changes include decreases in electrically evoked maximal force (Koryak, 1995), specific tension and maximal integrated electromyography (Berg et al., 1997; Dudley et al., 1992) along with increased submaximal electromyography (Berg et al., 1997) and neuromuscular junction dysfunction (Grana et al., 1996). Losses in muscle mass (Trappe et al., 2009), muscle strength (English et al., 2020, 2015) and muscle power (Trappe et al., 2009) impair functional performance (Miller et al., 2018; Mulavara et al., 2018) and negatively affect metabolic health (Hughson et al., 2016; Stein et al., 1994). Bone mineral density is reduced (Sibonga et al., 2015; Smith et al., 2015), and bone architecture is altered, as observed by changes in trabecular structure (Sibonga, 2013). Central interpretation of visual, vestibular and proprioceptive information is altered, in addition to sensorimotor derangements in perception, posture, gait, spatial orientation, lower limb kinematics, landing strategies after jumping and visual acuity during walking (English et al., 2019; Miller et al., 2018; Mulavara et al., 2010, 2018; Tays et al., 2021). Other physiological systems critically affected by spaceflight include the ocular (Wojcik et al., 2020) and immune systems (Crucian et al., 2018). Finally, sleep, a critical underpinning of all physiological function, is also dysregulated in the spaceflight environment (Albornoz‐Miranda et al., 2023). Collectively, these physiological alterations might impair both the health and the performance of crewmembers, particularly on long‐duration exploration missions to Mars.

To provide a theoretical framework for the physiological readiness required for exploration spaceflight, we looked at other tactical occupations with significant physical demands. In the last ∼15–20 years, a novel perspective has emerged in the fields of military, law enforcement and firefighting, in which these professionals are viewed and trained as tactical/occupational athletes (Sell et al., 2010; Sterczala et al., 2023). This new categorization stems from the notable similarities between these professions and competitive athletes with regard to the criticality of physical fitness and preparedness in occupational health and safety and operational success. We have applied this tactical athlete model to spaceflight crewmembers (Hackney et al., 2015). Furthermore, we have adopted a sport science approach to the preparation and monitoring of these tactical athletes.

In this review, we outline a set of new or enhanced monitoring modalities designed to quantify the physiological status of crewmembers. Next, we provide a brief overview of physiological countermeasures used in spaceflight to date and summarize requirements for the same in the austere and autonomous environment of a Mars exploration mission. Finally, given that Mars exploration missions will be the most salient example yet of the astronaut–athlete paradigm (Hackney et al., 2015), we describe physiological countermeasures that begin with targeted preflight training with the goal of launching well‐prepared crewmembers, in‐flight exercise hardware and individualized nutrition and sleep regimens to protect crew health and performance. Collectively, an integrated approach to physiological monitoring and countermeasures will maximize the likelihood of exploration mission success.

## 2 PHYSIOLOGICAL MONITORING

Physiological monitoring is a cornerstone of sport science. To facilitate optimal athletic development and, ultimately, performance, coaches and sport scientists carefully track a variety of internal and external physiological metrics to inform management of the fitness–fatigue paradigm to ensure that an athlete is adapting favourably to training stimuli and that he/she is on track for competition readiness. Athlete monitoring refers to systematic testing and assessment that includes both chronic and acute variables. Chronic monitoring involves the evaluation of adaptations that require weeks to discern, such as changes in muscle strength and aerobic fitness, and broadly informs the design and programming of training for individual athletes. In contrast, acute monitoring consists of weekly or even daily tracking of metrics, such as fatigue, recovery and neuromuscular preparedness, that inform acute readiness to train and/or perform (Suchomel et al., 2021). In exploration crewmembers, acute monitoring could provide data to inform decision‐making regarding the performance of mission tasks, particularly extravehicular activity (EVA). Good scores on monitoring tests would give mission planners and crewmembers confidence to proceed with scheduled activities, whereas depressed scores might suggest delaying or modifying a task or re‐assigning it to a different crewmember. Together, monitoring metrics are used to: (1) inform long‐term and daily exercise programming decisions; (2) assess readiness to perform; and (3) provide a baseline to inform rehabilitation and return to activity after illness or injury. Physiological monitoring of crewmembers before, during and after spaceflight provides crucial data to inform exercise programme design and mission architecture at both the macro and micro levels. Here, we present a variety of potential chronic and acute monitoring metrics that will facilitate crewmember health and safety and optimal mission performance.

### 2.1 Muscle strength and power

Isometric and isokinetic measures have high reliability and validity (Drouin et al., 2004; Feiring et al., 1990; Laughlin et al., 2009; Pincivero et al., 1997; Saenz et al., 2010; Symons et al., 2005) and have been the gold standard in sport rehabilitative fields for assessing muscle strength and readiness for return to play. Dynamometers to measure these variables have been used on the ground to measure pre‐ and postflight strength (English et al., 2015) and on the International Space Station (ISS) to measure in‐flight strength (e.g., the Muscle Atrophy Research and Exercise System; English et al., 2013); however, the mass and volume of such devices might be prohibitive for exploration spaceflight. Isometric variables can be determined from whole‐body or single‐joint movements via force plates or small dynamometers. Currently, the hardware most amenable to practical implementation for both testing and exercise during exploration spaceflight consists of low‐profile force plates integrated with the selected in‐flight exercise device, which will, ideally, include a bench for upper body testing and exercise. This hardware set‐up will provide an operationally relevant total‐body strength assessment, including metrics of isometric peak force, rate of force development and net impulse for a given duration (De Witt et al., 2018; Ishida, Bazyler, et al., 2023, Ishida, Suarez, 2023; Lum et al., 2020). A small, hand‐held dynamometer (e.g., Tindeq Progressor, Trondheim, Norway) can measure the same variables for grip and finger strength, in addition to other joints and movements, such as knee extension and flexion (Labott et al., 2022; Merry et al., 2021). Grip strength is uniquely relevant for spaceflight mission tasks because EVAs are conducted in pressurized suits; thus, grip strength and endurance are crucial for performance (Madden et al., 2017; Rajulu et al., 2009). The inclusion of force plates and a small, hand‐held dynamometer will facilitate reliable monitoring of characteristics such as lower body strength via the isometric mid‐thigh pull, upper body strength via the isometric bench press, and single‐joint strength via movements such as knee extension and hand/finger extension/flexion.

Muscle power can be assessed with similar hardware; however, because measuring work requires a change in the centre of mass (i.e., displacement), isometric tests do not provide power output but instead a related metric, the rate of force development. Force plates could, potentially, be used on the Martian surface for dynamic tests such as a static jump, countermovement jump or the Bosco jump test (Bosco et al., 1983). These tests determine power metrics, such as peak or average watts, and can be used to inform exercise programming and, potentially, to assess neuromuscular fatigue with additional contextual information. Anaerobic power can also be assessed via a cycle or rowing ergometer. For example, tests such as the Wingate cycle protocol are used to determine peak and average power over a 30 s maximum effort (Castaneda‐Babarro, 2021). A rowing ergometer could be used to quantify anaerobic capacity and average power over a similar short time period.

### 2.2 Aerobic capacity, ventilatory threshold and critical power

Aerobic capacity (peak O_2_ uptake) and markers of physiological thresholds, such as the aerobic threshold (closely corresponding to the first lactate threshold and first ventilatory threshold) and the anaerobic threshold (closely corresponding to the second lactate threshold and second ventilatory threshold), are staples of monitoring cardiovascular fitness for assessment of an athlete's adaptation to training and readiness for task performance, especially in endurance sports (Bisi et al., 2011; Bunc & Leso, 1993; Pallares et al., 2016). Underlying these systemic changes in oxygen consumption at submaximal (decreased consumption) and maximal (increased consumption) intensities, adaptations to endurance exercise include cardiac remodelling leading to increases in muscle thickness and chamber volume (Kleinnibbelink et al., 2022), increases in stroke volume, cardiac output, arteriovenous O_2_ difference, capillary density and mitochondrial density (Rivera‐Brown & Frontera, 2012), decreases in submaximal heart rate and small reductions in blood pressure centrally and peripherally.

Both aerobic capacity and submaximal threshold values provide insight into performance capacity, because many endurance sports require athletes to perform at a large fraction of maximal O_2_ consumption for extended periods of time (Coyle et al., 1988). After long‐duration spaceflight, reductions in the first ventilatory threshold are approximately double the losses in aerobic capacity, suggesting that submaximal work levels are a more sensitive metric of aerobic fitness, in addition to being better aligned with functional performance (English et al., 2020). Aerobic fitness corresponds to task performance in elite tactical fields (Maupin et al., 2018) and in ground‐based test subjects performing exploration mission tasks (Ryder et al., 2019; Sutterfield et al., 2019). Sutterfield et al. (2019) evaluated the aerobic capacity associated with successful performance of a simulated exploration mission surface traverse task. The surface traverse, performed at moderate intensity, elicited an O_2_ cost of 28.7 mL/kg/min and necessitated a peak O_2_ uptake of ≥35.0 mL kg min^−1^ and first ventilatory threshold ≥ 20.9 mL kg min^−1^ to ensure a high probability of success; reduced aerobic fitness rapidly increased the likelihood of task failure (e.g., a peak O_2_ uptake of <30.0 mL kg min^−1^ was associated with ∼80% likelihood of task failure). In addition to aerobic capacity and ventilatory threshold, critical power was also a significant predictor of task success. The hardware most amenable to the evaluation of aerobic capacity and ventilatory threshold during exploration spaceflight is likely to be a metabolic gas analyser akin to the portable pulmonary function system used on the ISS (Moore et al., 2014) paired with the selected in‐flight exercise device.

### 2.3 Sensorimotor and cognitive

Critical mission tasks will probably need to be completed during the first days and weeks after Mars landing. Because sensorimotor and cognitive functions, such as balance, coordination and mobility, are impaired after long‐term isolation and re‐introduction to gravity after long‐duration spaceflight (Miller et al., 2018; Mulavara et al., 2010, 2018), monitoring sensorimotor and cognitive function will be essential during Mars missions, particularly during the early post‐landing period.

Assessments such as the bimanual Purdue Pegboard Test for coordination, the functional mobility test for mobility, and cube rotation tests for cognitive–motor function have been used as part of a battery to assess sensorimotor and cognitive function during and/or after spaceflight (Tays et al., 2021). Although data from periodic tests will provide a high‐level view of sensorimotor and cognitive function, acutely monitoring crewmembers prior to demanding EVAs might be needed to ensure crewmember safety and mission success. Kim et al. (2018) developed a portable sensorimotor assessment tool (the Portable Sensorimotor Assessment Platform) to quantify changes in static posture, gait and lower‐limb ataxia. Composed of wireless inertial sensors, this or a similar device and protocol could be used acutely to evaluate sensorimotor readiness prior to demanding EVA. Daily questionnaires, such as the Short Recovery and Stress Scale, have been validated and used with athletic populations to assess fatigue and readiness to perform. The Short Recovery and Stress Scale spans two dimensions and eight items (four items per dimension): physical performance capability, mental performance capability, emotional balance and overall recovery (recovery dimension); and muscular stress, lack of activation, negative emotional state and overall stress (stress dimension). This questionnaire is inobtrusive and might be useful for exploration spaceflight (Flynn et al., 2017; Kolling et al., 2019; Travis et al., 2019).

### 2.4 Heart rate variability

Heart rate monitoring has become an increasingly integrated aspect of athlete monitoring as technology has improved (Buchheit, 2014; Tomes et al., 2020). Previously, athletes had only a basic pulse rate to monitor acute training, training adaptations and recovery (Bellenger et al., 2016; Buchheit, 2014). Now, with more sophisticated monitors, heart rate variability (HRV) has emerged as a viable monitoring aid (Tomes et al., 2020). Analysis of HRV evaluates alterations in the autonomic nervous system, providing an assessment of parasympathetic and sympathetic nervous system balance (McEwen & Wingfield, 2003; Tomes et al., 2020). These data can be used to identify increased fitness and readiness and, conversely, overreaching and maladaptive stressors. Many variations exist on how to measure HRV; however, the most common approach evaluates low‐ and high‐frequency activity, the ratio of the two, and spectral and root‐mean‐square of successive differences temporally (Buchheit, 2014; Shaffer & Ginsberg, 2017). To gain the most insight from HRV monitoring, it is crucial to standardize data‐collection methods and to establish individual, preflight HRV baselines during periods of both lower and higher training demand (Buchheit, 2014). Monitoring HRV immediately upon waking, in the last stage of deep or slow‐wave sleep and/or post‐activity appears to be most effective when both common spectral and temporal monitoring is used (Buchheit, 2014; Tomes et al., 2020). Although the nuances of HRV monitoring are beyond the scope of this review, numerous studies in monitored occupational and tactical athletes have shown the sensitivity of HRV to detect important physiological changes, namely, increased aerobic fitness in firefighters with favourable changes to HRV (Porto et al., 2019) and high levels of stress in post‐combat scenarios and during shift participation in military personnel (Sanchez‐Molina et al., 2018) and first responders (Kaikkonen et al., 2017). Performance for tactical athletes includes both physical and cognitive components; alterations in HRV metrics are associated with changes in cognitive performance (Tomes et al., 2020), including decreased working memory (Delgado‐Moreno et al., 2017; Johnsen et al., 2012). Postexercise and resting HRV, when combined with measures of heart rate recovery, in athletic populations mark favourable adaptations and increased fitness (Bellenger et al., 2016). Careful monitoring of HRV will provide insight into the fitness of crewmembers, signal periods of overreaching and high stress, and help to inform performance readiness.

## 3 PHYSIOLOGICAL COUNTERMEASURES

To protect the health of crewmembers and enhance the likelihood of mission success via preserved physical performance, physiological countermeasures have been implemented throughout human spaceflight (Scott et al., 2019). These interventions have evolved with increased scientific understanding and technological advancements and vary somewhat based on mission duration. Current exercise countermeasure hardware on the ISS includes the following: a cycle ergometer that provides a maximal power output of 350 W (CEVIS; Cycle Ergometer with Vibration Isolation System); a treadmill that allows a maximum velocity of 19.3 km/h (Treadmill 2; T2); and a resistance exercise device that provides ≤273 kg of loading (the Advanced Resistive Exercise Device; ARED) (English et al., 2019). We recently reported that this suite of exercise hardware, coupled with programming of high‐intensity training, better protected muscle function, bone mineral density and cardiorespiratory fitness during long‐duration spaceflight in comparison to legacy hardware and protocols, although losses in key outcomes persisted (English et al., 2020). Unfortunately, the ISS exercise hardware suite consists of high‐mass and large‐volume devices, which are untenable for an exploration mission with a small capsule (English et al., 2019).

Upcoming short‐duration Artemis missions, which include lunar orbit and surface operations on the Moon, will use a single in‐flight exercise device for both aerobic and resistance exercise (Boyer et al., 2023). At the same time, versions of this inertial flywheel device have been shown to prevent or mitigate bed rest‐induced decrements in muscle mass, muscle strength, muscle power, bone mineral density and cardiorespiratory fitness (Alkner et al., 2016; Belavy et al., 2017; Ploutz‐Snyder et al., 2018; Rittweger et al., 2005). To date, a flywheel has not served as the primary exercise device during a spaceflight mission. Practical operational factors, in addition to physiological stimulus limitations, might also influence the effectiveness of a single, capsule‐based flywheel, including: (1) the necessity of sharing a single piece of hardware between a four‐person crew for four to six daily workouts that total 4–6 h of hardware use per day (English et al., 2020; Loehr et al., 2015); (2) the need for adequate CO_2_ removal by the environmental system of the capsule to facilitate this many hours of exercise, some of which will be at high intensity; (3) the absence of an ambulatory countermeasure component (i.e., a treadmill), which might be crucial to protect sensorimotor function, neuromuscular coordination (Macaulay et al., 2021), skeletal muscle (Trappe et al., 2009) and bone mineral density; and (4) the use of a rowing ergometer, integrated in the flywheel device, as the sole aerobic exercise modality.

Mars missions are still notional but will probably include all the aforementioned obstacles related to spaceflight exploration in a small capsule, plus additional challenges particular to deep space exploration. One prominent mission scenario consists of a ∼6‐month transit to Mars, an 18‐month surface stay and a 6‐month return flight, for a total mission duration of ∼2.5 years (Clement et al., 2020). During the two 6‐month intravehicular transit periods, four crewmembers will be confined in a small capsule, where they will eat, sleep and exercise. Once on the Martian surface, long communication delays of 5–20 min will demand increased crew autonomy. Unlike current ISS operations, which are conducted in microgravity, terrestrial Martian exploration will occur in three‐eighths gravity; given the mass of current exploration spacesuit designs, it is possible that the system weight of a suited crewmember on Mars will approach the crewmember's 1*g* body weight. Physiological demand for Mars surface operations will be greater than corresponding ISS and lunar surface activities (Ryder et al., 2019; Sutterfield et al., 2019). Notional mission architecture calls for physically demanding activities, such as capsule egress, spacesuit donning and doffing, habitat construction, terrestrial surface ambulation and exploration, hill climbing, equipment deployment, vehicle operation and geological sampling; off‐nominal tasks include rescue of an incapacitated crewmember (Coan, 2020). Although it is likely that robots and/or exoskeletons will be available to assist crewmembers with the most physically demanding exploration tasks, crewmembers should, nevertheless, be prepared to complete most of these tasks unassisted, because over‐reliance on mechanical support could prove problematic in off‐nominal and contingency scenarios.

**FIGURE 1:**
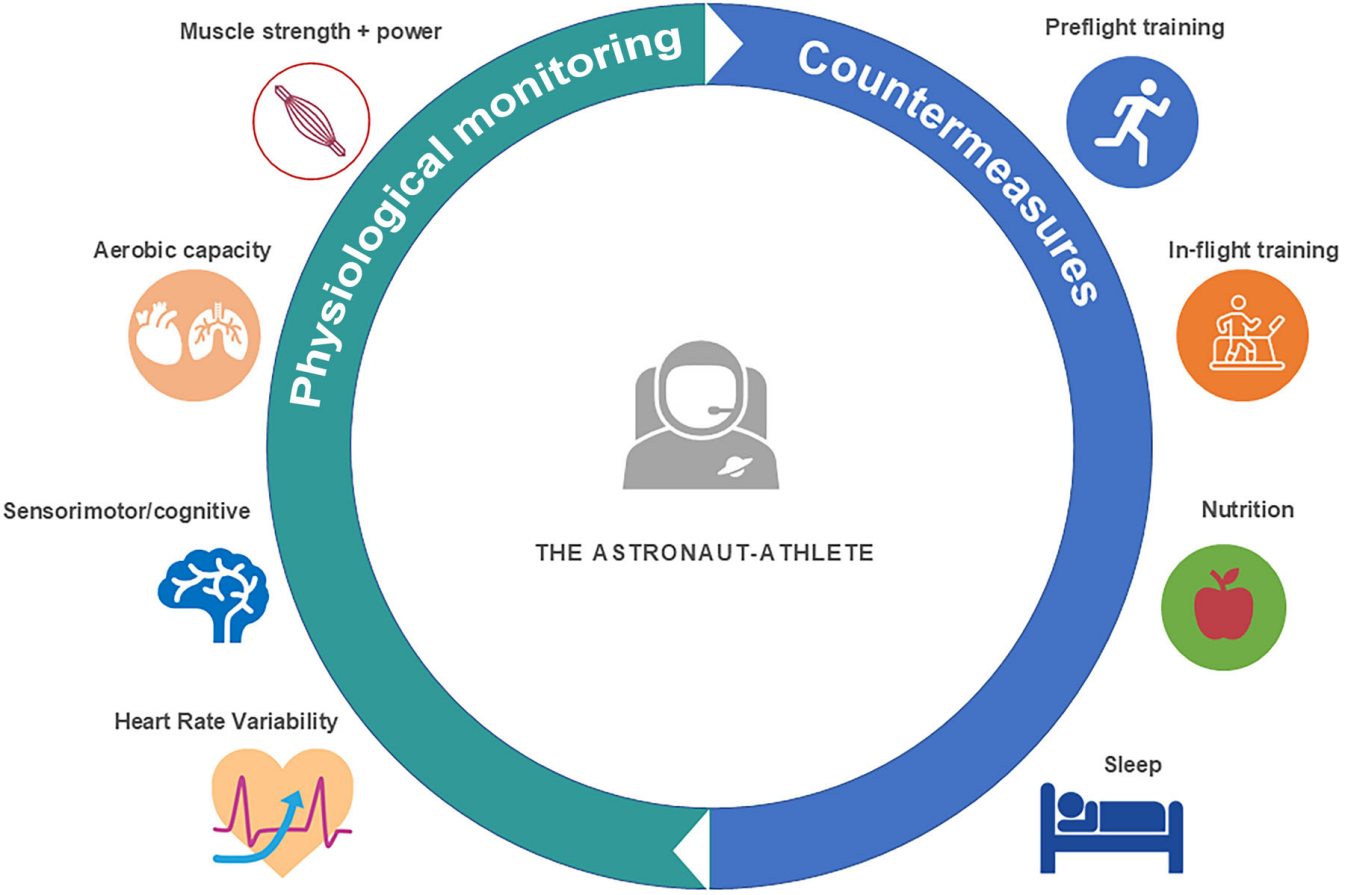
Physiological monitoring and countermeasures for the astronaut–athlete on Mars exploration missions.

With the view of long‐duration exploration crewmembers as tactical athletes, we apply a variety of well‐characterized principles, such as periodization of training, to their preparation (Cunanan et al., 2018; Stone et al., 2021). Additional principles of exercise training, such as progressive overload and movement specificity, are considered in most models, although they would probably need to be adapted to address the specific nature of spaceflight. Periodization models typically consider training an athlete over a period of 1 year or more, from development to their most important competition. A Mars mission consisting of several months of spaceflight followed by landing and planetary objectives allows for a development period prior to launch and during spaceflight and a ‘competition‐like’ period while on the Mars surface. Following a periodization model in‐flight would prioritize the development of these qualities at critical time points (e.g., landing and EVA) through progressively loaded resistance training and fatigue management (Sands et al., 2012). We focus on exercise training but also discuss emerging approaches towards nutrition and sleep (Figure 1).

### 3.1 Preflight training

Preflight physiological training is a crucial and, perhaps, historically neglected training phase; neglected owing to the travel and mission‐specific training obligations of crewmembers. Busy schedules notwithstanding, the preflight mission phase affords hardware availability and regular, often direct, individualized training, during which physiological weaknesses are most amenable to remediation. This period also provides an excellent opportunity to individualize the in‐flight exercise prescription and familiarize crewmembers with the equipment, protocols and habits that will be most useful during spaceflight. Most long‐duration crewmembers lose muscle strength during spaceflight; the few who maintain or even gain strength during flight are on the low end of the strength spectrum (English et al., 2015). It is likely that during preflight physiological preparation, these crewmembers trained with relatively low volume, low intensity, or both, and thus, the performance of regular in‐flight exercise countermeasures was sufficient to maintain or even increase their low level of preflight fitness. Regardless of the small increases in strength that these individuals realized during spaceflight, they remained among the weakest long‐duration crewmembers (English et al., 2015). To prepare crewmembers for the sensorimotor challenges of spaceflight, Bloomberg et al. (2015) outlined a preflight sensorimotor training regimen to enhance the adaptability of crewmembers to new gravitational environments. Thus, the most impactful and easily implemented approach to optimal in‐flight fitness and mission performance is a robust preflight training period that launches exploration crewmembers with high levels of physiological readiness (Ryder et al., 2019; Sutterfield et al., 2019).

### 3.2 In‐flight training

Resistance training modalities in spaceflight must rely on tension, pressure or inertia to provide resistance, because the typical resistance training attributable to gravity encountered on the Earth's surface is negated by the effects of microgravity. Implements such as resistance bands, hydraulic/vacuum systems or flywheel inertial trainers can be used to provide resistance for a training programme. For example, the current ISS resistance exercise device, ARED, uses two vacuum cylinders and inline flywheels to provide constant resistance and inertial resistance, respectively (Loehr et al., 2015, 2011). To provide optimal protection for skeletal muscle and bone in an unloaded environment, resistance exercise hardware should incorporate: (1) a high loading capability (English et al., 2015; Loehr et al., 2011; Smith et al., 2012); (2) resistance with a real or simulated inertial component (Amonette et al., 2004; English et al., 2019; Schneider et al., 2003); and (3) resistance during the eccentric phase of the movement that is equal to or greater than the resistance provided during the concentric phase (English et al., 2014).

Hardware for cardiorespiratory training is relatively straightforward, because a variety of ergometers (e.g., treadmill, cycle ergometer, rowing ergometer) can supply the necessary resistance to stimulate the aerobic and glycolytic energy systems. However, this does not consider the multifaceted effects of aerobic training on other physiological systems, such as the vestibular, sensorimotor and musculoskeletal systems. Current understanding is that ambulation provides a unique stimulus to these systems that cycle and rowing ergometers do not (English et al., 2019; Macaulay et al., 2021; Sibonga et al., 2019, 2007); greater treadmill use on the ISS is even associated with enhanced protection of muscle mass and muscle strength (Trappe et al., 2009), properties that are typically more associated with resistance exercise. On the ISS, crewmembers have always had access to a treadmill; however, current plans for short‐duration lunar missions do not include an in‐flight treadmill. The absence of ambulatory exercise (i.e., a treadmill) during a lengthy Mars transit flight will probably be deleterious to crew performance on the Martian surface (Miller et al., 2018, 2010; Mulavara et al., 2010, 2018). To elucidate the criticality of in‐flight treadmill exercise better, a study is underway to evaluate postflight physiological outcomes and functional performance in ISS crewmembers using exploration‐focused exercise hardware with no treadmill exercise during long‐duration ISS missions (Varanoske et al., 2023). Macaulay et al. (2021) have proposed a four‐pronged countermeasure approach to protect sensorimotor and, specifically, proprioceptive function during exploration spaceflight comprising: (1) axial body loading (potentially including lower‐body negative pressure; Petersen et al., 2019); (2) postural/proprioceptive challenge; (3) tactile input; and (4) sensory feedback. Exercise, in general, is an excellent countermeasure to spaceflight and confinement‐induced cognitive decline (Schneider et al., 2010).

An additional tool for consideration for in‐flight exercise countermeasures is blood flow restriction exercise. Blood flow restriction is used with resistance exercise and consists of partial or complete occlusion of the blood flow to exercising extremities during either the working portion of a set or both the working and rest periods of several consecutive sets. Although the loads lifted during blood flow restriction exercise are much lower than those of traditional resistance exercise, increases in muscle mass and muscle strength are comparable (Moore et al., 2004; Takarada et al., 2002; Wang et al., 2023). Blood flow restriction exercise has been used in older adults (Cook et al., 2017; Karabulut et al., 2010), in postsurgical rehabilitation (Roman et al., 2023) and in ground‐based disuse models (e.g., unilateral limb suspension and bed rest), where it has demonstrated efficacy to mitigate unloading‐induced losses in muscle mass and strength (Cook et al., 2010; Hackney et al., 2016).

### 3.3 Nutrition

Adequate nutrition is a vital component of human spaceflight exploration. Historically, reduced in‐flight energy intake led to decreases in body mass, particularly fat‐free mass, during long‐duration spaceflight (Smith et al., 2005; Zwart et al., 2014). Combined with poor vitamin D status, hypocaloric intake also contributed to significant loss of bone mineral density during early ISS missions (Sibonga et al., 2008; Smith et al., 2005). Conversely, eucaloric energy intake coupled with nominal vitamin D status and robust resistance exercise hardware (Loehr et al., 2011) prevented loss of bone mineral density in long‐duration ISS crewmembers (Smith et al., 2012).

Energy expenditure during spaceflight also plays an important role in physiological homeostasis. Bourdier et al. (2022) monitored energy expenditure, energy intake, body composition, activity levels and their relationships to each other in long‐duration ISS crewmembers both prior to and during flight. Crewmembers who maintained their preflight total energy expenditure during flight saw positive body composition changes (i.e., fat‐free mass maintenance and fat mass loss); conversely, crewmembers with reduced in‐flight total energy expenditure experienced negative body composition changes (i.e., losses in fat‐free mass and increases in fat mass). These differential changes occurred within the context of both groups incurring insignificant body mass losses and highlight the potent protective effects on body composition of both formal exercise countermeasures and non‐exercise physical activity. A note in reference to our previous comments on the importance of preflight fitness and in‐flight treadmill exercise: the authors reported positive associations between preflight body composition, preflight activity levels (specifically more time running or engaging in vigorous physical activity) and in‐flight treadmill usage with in‐flight body composition changes.

After the attainment of eucaloric energy intake and nominal vitamin D status, adequate protein intake is the third pillar in a well‐composed spaceflight diet. Ground‐based unloading studies demonstrate that insufficient protein intake is deleterious to the maintenance of fat‐free mass (Ferrando et al., 2010; Paddon‐Jones et al., 2004; Stuart et al., 1990). Although mean protein intake on the ISS is greater than the US recommended dietary allowance historically (Smith et al., 2005), there remains the possibility of optimizing protein intake on an individual crewmember basis. Indeed, a recent position stand on nutrition for athletes emphasizes the importance of nutritional programming based not only on the needs of the individual athlete, but also on the variation of those needs in different training periods and environments, that is, ‘periodized nutrition’ (Thomas et al., 2016). In athletes, the recommended daily protein intake is 1.2–2.0 g (kg body weight) ^−1^ day^−1^, with benefits for even greater intakes in certain situations (Thomas et al., 2016). Ideally, dietary protein should be consumed over three or more meals throughout the day, with ∼0.4–0.6 g (kg body weight) ^−1^ per meal, and in close temporal proximity to exercise training (Thomas et al., 2016). Ground‐based models of spaceflight unloading suggest that the above guidelines might also be applicable to spaceflight (Brooks et al., 2008; Ferrando et al., 2010; Paddon‐Jones et al., 2004). Specific scenarios in which higher protein intakes have been shown to exert protective effects include periods of negative energy balance (Carbone et al., 2019; Hiroux et al., 2022; Longland et al., 2016; Pasiakos et al., 2013) and during reduced physical activity (English et al., 2016). Protein intake should be prescribed within the overall context of individual energy intake (i.e., eucaloric vs. hypocaloric) and physical activity (i.e., reduced vs. typical or increased). For an extensive discussion of other nutritional issues relevant to spaceflight exploration, including food systems and the potential protective effects of nutrition on neurocognitive function, readers are referred to the excellent review by Zwart et al. (2021).

### 3.4 Sleep

Sleep is a critical underpinning of health and, in concert with exercise and nutrition, forms the foundation upon which physiological and cognitive performance rests (Charest & Grandner, 2020). Spaceflight crewmembers experience reduced sleep duration and altered sleep architecture, such as less rapid eye movement sleep and increased rapid eye movement latency (Piltch et al., 2024; Stickgold & Hobson, 1999). Circadian misalignment is more prevalent than on Earth and is associated with shorter sleep duration and increased use of sleep‐promoting medication (Flynn‐Evans et al., 2016). Impaired sleep during spaceflight elicits increases in psychomotor response speed, stress, and somatic behavioural states such as physical exhaustion and mental fatigue (Jones et al., 2022). Challenges to sleep during Mars transit will probably be similar to those documented during low Earth orbit and short‐duration flights, although Martian sojourn will introduce additional complexities, including circadian alterations owing to the 24 h, 39 min Martian day (sol) and dust storms that block sunlight (Albornoz‐Miranda et al., 2023).

Wu et al. (2018) outline a series of proposed countermeasures to the deleterious effects of spaceflight on sleep, including an improved sleep environment, improved work–rest schedules, pharmacological regimens, improved lighting, psychological support, crew selection and training, and Tai Chi training. Lighting is probably the most researched topic in this list, with exposure to blue light being shown to suppress melatonin production and circadian rhythm (Tosini et al., 2016). Mars missions will need to use these or other innovative strategies to optimize the health and performance of exploration crewmembers.

## 4 CONCLUSIONS

Multisystem physiological monitoring and countermeasures in spaceflight have been used for >50 years and provide important foundations for future requirements for exploration missions to Mars. However, enhanced physiological monitoring will be required to optimize astronaut health and performance. Moreover, physiological interventions that span preflight and in‐flight settings and combine new and individualized nutrition and sleep regimens will be needed to protect crewmember health and performance. We advocate a sport science approach to physiological monitoring and countermeasures for the astronaut–athletes serving as crewmembers on Mars exploration missions.

## AUTHOR CONTRIBUTIONS

Kirk L. English and Jessica M. Scott conceived the original conceptual approach to the manuscript. Luke DeVirgiliis, Nicholas J. Goode, Kurt W. McDowell, Kirk L. English, Robert Novo, Virina Botros, Ginika Agwu, Jessica M. Scott, and Lori L. Ploutz‐Snyder critically revised and contributed to the revised manuscript. All authors approved the final manuscript and agreed to be accountable for all aspects of the work in ensuring that questions related to the accuracy or integrity of any part of the work are appropriately investigated and resolved. All persons designated as authors qualify for authorship, and all those who qualify for authorship are listed.

## CONFLICT OF INTEREST

None declared.
